# People with symptoms of depression and those at significant risk of suicide show differences in their personality profile and sense of meaning in life

**DOI:** 10.3389/fpsyt.2025.1508791

**Published:** 2025-02-05

**Authors:** Kacper Deska, Grzegorz Mirocha, Bartłomiej Bąk, Anna Mirgos-Wierzchowska, Marcin Kosmalski, Monika Różycka-Kosmalska, Tadeusz Pietras

**Affiliations:** ^1^ Student’s Scientific Association Clinical Pharmacology, Medical University of Lodz, Lodz, Poland; ^2^ Department of Biostatistics and Translational Medicine, Medical University of Lodz, Lodz, Poland; ^3^ Department of Child and Adolescent Psychiatry, Rehabilitation Hospital - Konstancin-Zdrój Health Resort, Konstancin Jeziorna, Poland; ^4^ Department of Clinical Pharmacology, Medical University of Lodz, Lodz, Poland; ^5^ Department of Electrocardiology, Medical University of Lodz, Lodz, Poland

**Keywords:** depressive symptoms, suicide risk, personality, sense of life, medical students

## Abstract

**Introduction:**

Medical students are exposed to various stressors. Among the many factors that determine the possibility of a mental crisis, there is also a personality profile and a sense of meaning in life.

**Materials and methods:**

Sets of anonymous surveys were distributed among medical students of different years studying at the Medical University of Lodz. The set of surveys included a sociodemographic survey, Beck’s Depression Inventory version II (BDI-II), the NEO Five Factory Inventory (NEO-FFI), Reker’s Life Attitude Profile - Revised questionnaire (LAP-R), Osman’s Suicidal Behavior Questionnaire (SBQ-R).

**Results:**

The study cohort comprised of 276 students (mean age 21.7 years). According to the BDI-II, 79 participants (28.4%) were identified as having depressive symptoms. Additionally, 80 participants (28.9%) were assessed to be at significant risk of suicide according to the SBQ-R scale. Based on the results of these questionnaires, we identified four groups: 1. Participants with depressive symptoms (D). 2. Participants with suicide risk (SR), 3. Participants with both depressive symptoms with suicide risk (D and SR), 4. A control group. Students from D and D and SR groups, exhibited higher neuroticism scores compared to those with suicide risk alone (SR) and the control group. In terms of extroversion, the control and SR groups scored higher compared to the D with SR group. Participants with SR and those with D and SR had higher openness scores compared to the D and control groups. D and SR group obtained statistical lower score then control group in the terms of conscientiousness. In life control score, participants in D and D with SR group has significant lower score then SR and control group. The conditions: personal meaning index and life attitude balance in the control group achieved significantly higher values compared to all other groups.

**Conclusion:**

People with depressive symptoms, suicide risk and both of these variables simultaneously differed in terms of personality profile and components influencing the meaning of life.

## 1 Introduction

Medical students are a diverse group that is exposed to specific stressors during their education: those resulting from the course and characteristics of education, such as the burden of the amount of knowledge to be acquired, frequent knowledge tests, strict study regulations or mobbing from other members of the academic community; those of a moral and ethical nature, such as contact with suffering and death or the burden of control over the patient’s therapeutic process; of social and family nature, such as high expectations from family members or the society, as well as a change in the environment of everyday functioning (1, 2).

The results of research on the prevalence of depressive disorders in this group have varied considerably to date, which is related to methodological differences and the research tools used (3–5). A meta-analysis by Puthran et al. demonstrated that the prevalence of depressive disorders in the group of students may reach 28.0%, and in Europe 20% of students. The highest percentage of depressive disorders – 33.5% –was recorded in the group of first year students, and decreased with subsequent years of study (6). Similar results are presented in a meta-analysis by Rotenstein and co-workers, in which the prevalence of depressive symptoms among students reached 27.2% (7).

Most suicides are related to psychiatric diseases, with depressive symptoms, substance use disorders and psychosis being the most relevant risk factors (8, 9). The risk of suicide has been estimated at 5–8% for several mental disorders, such as depression, alcoholism and schizophrenia (10). The research shows that there is an increased risk of suicide in the group of medical students. Based on the meta-analysis by Rotenstein et al., the summary prevalence estimates ranged across assessment modalities from 7.4% to 24.2% (7).

Exposure to external stressors among different medical students is similar, yet only a fraction of them experience a mental crisis. According to research, one of the factors that influences susceptibility to a mental crisis is personality structure. Due to the complexity of the construct that personality is, as well as numerous concepts of its development and description, it is impossible to create a coherent model of the pathogenesis of mood disorders in this approach. Nevertheless, the structural elements of personality and differences in the severity of individual personality traits are currently considered to be one of the risk factors for depressive disorders (11–13). Many psychometric tools have been developed for research purposes. Assessment of personality structure involves isolating personality traits, for which questions are created. Then the participant answers the questions, and the researcher tallies the results and describes the personality structure of the person being examined. Depression can have a two-way effect on the perception of meaning in life, the feeling of control over one’s life and the sense of purpose of the actions performed (14, 15). Studies have clearly shown that depressive symptoms correlates negatively with a sense of meaning in life (16).

The aim of this study was to estimate the prevalence of depressive disorders and suicidal risk in a group of medical students at one of the Polish universities, taking into account personality traits according to the Big Five theory and the sense of meaning in life. An additional goal of our work was to find differences between the group of people with depressive symptoms, suicidal risk and both of these variables simultaneously in terms of personality traits and components influencing the meaning of life. Finding such differences provides further opportunities in attempts to identify patients with depressive symptoms in terms of the possibility of suicidal thoughts based on personality traits. On the other hand, determining which of the traits influencing the sense of meaning in life is a problem provides the possibility of targeted psychotherapy.

## 2 Materials and methods

### 2.1 Participants

The participants recruited were students at the Medical University of Łódź, Poland. Participation in the study was voluntary; all students gave written, informed consent and did not receive compensation for their participation.

The inclusion criteria were as follows: student status at the university; over 18 years of age; written informed consent.

The exclusion criteria included individuals who did not give their written consent to participate in the study or were otherwise unable to complete the survey on their own.

### 2.2 Measures

Each questionnaire was completed by the participants themselves on paper. During the completion of the questionnaires, full anonymity of the participants was ensured.

All participants provided their primary socio-demographic data such as age, gender, year of study, relationship status, previous psychiatric diagnoses and treatment.

#### 2.2.1 Depressive symptoms

The severity of depressive symptoms was assessed using the Beck’s Depression Inventory version II (BDI-II), a validated questionnaire with 21 items, each rated on a scale of 0–3 by the participant (17, 18). In this scale the internal consistency was described as around 0.9 and the retest reliability ranged from 0.73 to 0.96 (19). Severity of Depression Symptoms according to the results obtained:

- 14 – 19 – mild depressive symptoms.
- 20 – 28 – moderate depressive symptoms.
- 29 – 63 – severe depressive symptoms.

#### 2.2.2 Personality structure

To assess the personality structure, the validated Polish version of the NEO Five Factory Inventory (NEO-FFI) by Paul T. Costa Jr. and Robert R. McCrea was used (20). Validation to the Polish population was carried out by the Psychological Testing Laboratory of the Polish Psychological Association based in Warsaw (21). It evaluates 5 components of human personality: Neuroticism, Extroversion, Openness to Experience, Agreeableness and Conscientiousness on the basis of 60 questions (12 for each trait), and the respondent answers each question on a scale from 1 to 5. In this questionnaire, 1 means that the participant does not agree with the statement, 5 that he or she completely agrees, and 3 that it is a neutral statement with regard to the given person (21). In this questionnaire, the internal consistency for the individual components ranges from 0.69 to 0.86 (20).

#### 2.2.3 Sense of meaning of life

The assessment of the sense of meaning in life was carried out using the Polish version of Life Attitude Profile – Revised (LAP-R) by Gary T. Reker (22). The questionnaire consists of 48 questions, which make up 6 simple scales: Purpose, Coherence, Life Control, Death Acceptance, Existential Vaccum, Goal Seeking and 2 complex scales: Personal Meaning Index and Life Attitude Balance. The respondents rate the answers on a scale from 1 to 7. In this questionnaire, 1 means that the participant does not agree with the statement, 7 that he or she completely agrees, and 4 that it is a neutral statement with regard to the given person (22). The consistency score for Cronbach’s LAP-r Alpha scale was 0.73 (23).

#### 2.2.3 Suicidal risk

To assess suicidal risk, A. Osman’s Suicidal Behavior Questionnaire (SBQ-R) in Polish version was used (24, 25). It consists of 4 questions, 3 of which concern suicidal behaviors and tendencies in the past, and 1 about the desire to attempt suicide in the future. According to the Polish adaptation, the cut-off score of 7 points was assumed to represent a high suicidal risk (24). Internal consistency assessed using Cronbach’s α was 0.83 (24). Participants who scored above 6 and above 1 in first item were considered to be at significant risk of suicide.

### 2.3 Data collection

The data were collected from October 2022 to January 2023. An anonymous questionnaire was distributed to students after compulsory class by undergraduate researchers. The classes after which participants received surveys were selected randomly. Volunteers were invited to complete the study. The initial questions concerned the participants’ demographic data. Then they completed the Neo-FFI, LAP-R, SBQ-R and BDI-II. After collecting a sufficient number of correctly filled-out questionnaires, the data were analyzed.

### 2.4 Statistical analysis

The normality of the distribution was assessed using the Shapiro-Wilk test. Due to the non-normal distribution of most variables, continuous data are reported as median values with interquartile ranges (IQR), unless otherwise specified. Spearman’s correlation coefficient was employed to evaluate the correlations between questionnaires. Correlation coefficients (ρ) were interpreted as follows: ρ > 0.8 indicated a very strong correlation, 0.6 < ρ ≤ 0.8 indicated a strong correlation, 0.4 < ρ ≤ 0.6 indicated a moderate correlation, 0.2 < ρ ≤ 0.4 indicated a weak correlation, and ρ ≤ 0.2 indicated a very weak correlation. Differences in questionnaire scores between two groups were analyzed using the Mann-Whitney U test, while differences among more than two groups were assessed using the Kruskal-Wallis test. For significant results from the Kruskal-Wallis test, *post-hoc* analysis was performed using the Conover-Iman test with Holm-Bonferroni correction. Categorical variables were analyzed using the Pearson chi-squared (χ²) test, with Yates’ correction applied when any subgroup had fewer than 15 participants. If any analyzed subgroup had fewer than 5 participants, Fisher’s exact test was utilized. All statistical analyses were conducted using Python version 3.11.5 with the SciPy package (version 1.14.1).

## 3 Results

### 3.1 Group characteristics

The study cohort comprised of 276 (173,62.7% were female) medical students with mean age at participation 21.7 years (SD = 2.7). The most of participants were in their first year of studies (79, 28.6%), followed by those in their second (58, 21%), fifth (51, 18.5%), third and sixth (35, 12.7%), and fourth (18, 6.5%) years. Other socio-demographic characteristics of the participants enrolled in the study are presented in **Table 1**.

**Table 1:** The characteristics of participants included in the analysis.

Characteristics	Total
Number of participants	276 (100)
Age (mean, SD)	21.7 (SD = 2.7)
Gender	Men: 103 (37.3)
	Women: 173 (62.7)
Year of studies First Second Third Fourth Fifth Sixth	79 (28.6) 58 (21.0) 35 (12.7) 18 (6.5) 51 (18.5) 35 (12.7)
Marital status Single In relationship Married Missing	147 (53.3) 118 (42.8) 10 (3.6) 1 (0.4)

Data are shown as frequencies N (%) unless otherwise specified.

### 3.2 Descriptive analysis

In our study, 37 participants (13.4%) reported having a diagnosed mental illness, of which 19 (51.4%) suffered from depression. Among those with a mental illness, 29 participants (78.3%) were receiving pharmacological treatment. One participant diagnosed with depression and one with another unspecified mental illness did not disclose their medication status.

In the entire cohort, the median BDI-II score was 7 (IQR=12), while the median SBQ-R score was 4 (IQR=4). According to the BDI-II scale, 79 participants (28.4%) were identified as having depressive symptoms, with 32 (11.5%) classified as mild, 24 (8.6%) as moderate, and 23 (8.3%) as severe cases. Additionally, 80 participants (28.9%) were assessed to be at significant risk of suicide according to the SBQ-R scale, 25 (31%) of them was classified as high suicide risk (SBQ-R>10 points).

Within the subset of participants previously diagnosed with depression, 7 (36.7%) did not score positively on the BDI-II scale, and 3 (15.8%) did not score positively on the SBQ-R scale. Based on the results of these questionnaires, we identified four groups:

1. Participants with depressive symptoms, who scored positively only on the BDI-II scale or had a previous diagnosis of depression (D).
2. Participants with suicide risk (SR), who scored positively only on the SBQ-R scale.
3. Participants with both depressive symptoms and suicidal risk (D and SR), who scored positively on both the BDI-II and SBQ-R scales.
4. A control group consisting of other participants.

**Figure 1:**
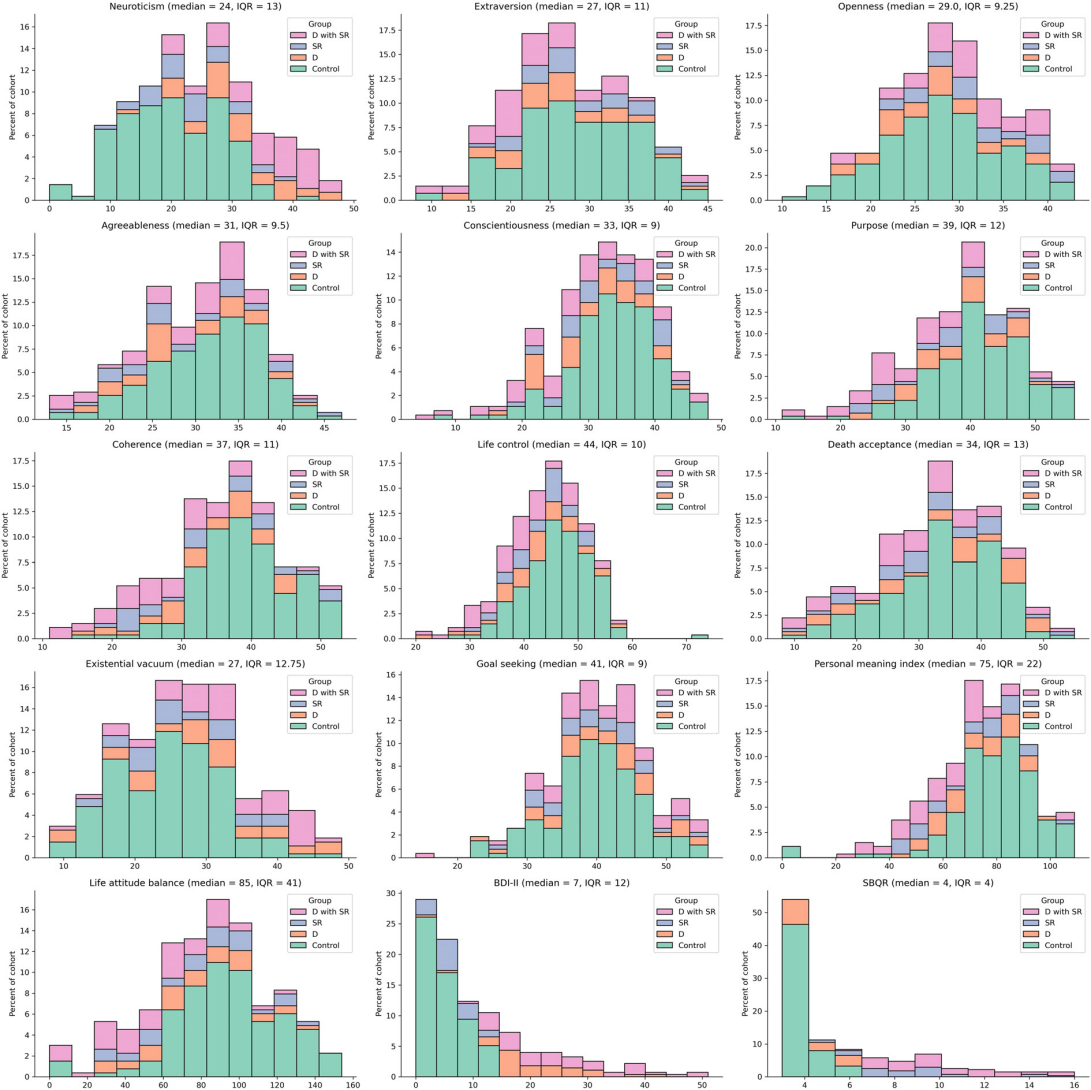
The histograms represents distribution of NEO-FFI, LAP-R, BDI-II, and SBQ-R results.

In summary, the D group comprised 37 participants (13.4%), the SR group included 31 participants (11.2%), the D with SR group consisted of 49 participants (17.7%), and the Control group was made up of 159 participants (57.6%). The NEO-FFI, LAP-R, BDI-II and SBQ-R results are presented on the **Figure 1**.

### 3.3 Factors associated with BDI-II and SBQ-R score

To assess potential factors associated with depressive and suicide risk, we first compared socio-demographic data with scores from the Beck Depression Inventory-II (BDI-II) and the Suicide Behavior Questionnaire-Revised (SBQ-R). Our analysis revealed that females had a significantly higher SBQ-R score than males (median = 5, IQR = 4 vs. median = 4, IQR = 3; p = 0.03); however, the difference in the frequency of positive responses was not statistically significant (20.2% vs. 8.6%, p = 0.1). In contrast, the BDI-II scores did not show a statistically significant difference based on gender (p = 0.2).

Furthermore, first-year students exhibited higher BDI-II scores compared to third-year (12 (15) vs. 4 (7); padj < 0.001) and fifth-year students (12 (15) vs. 3 (9.5); padj < 0.001), and they were more likely to score positive on the BDI-II scale (46% vs. 9%, and 46% vs. 14%, p < 0.001, respectively). In the SBQ-R scale, only the difference between first and third-year students was significant (5 (5) vs. 3 (1); p < 0.001), with first-year students also scoring positively on the SBQ-R scale more frequently (35% vs. 14%; p = 0.0344).

**Figure 2:**
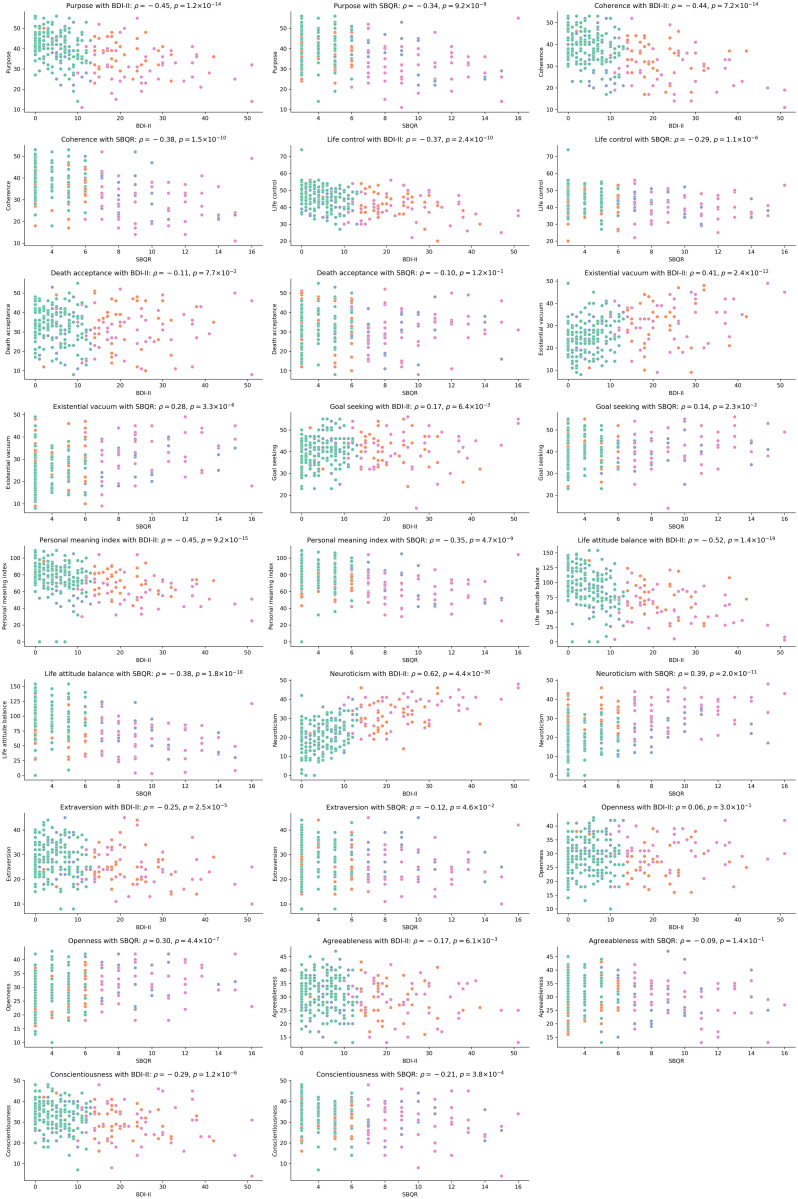
Represents scatterplots with results of correlation analysis. The y-axis represents scores in NEO-FFI or LAP-R, whereas x-axis represents BDI-II or SBQ-R score.

Notably, there were no significant differences observed between relationship status and either the BDI-II or SBQ-R scores. We further explored potential links between traits measured by the NEO-FFI, LAP-R, and the BDI-II/SBQ-R scales. Our correlation analysis revealed strong positive correlations between the BDI-II and neuroticism, alongside moderate negative correlations between the BDI-II and variables such as purpose, coherence, life attitude balance, and the personal meaning index. We also found moderate positive correlations between the BDI-II and the existential vacuum. Weak positive correlations were identified between SBQ-R scores and neuroticism, openness, and the existential vacuum, while negative correlations were found between SBQ-R and coherence, life attitude balance, life control, purpose, conscientiousness, as well as between BDI-II and conscientiousness and extroversion. Very weak positive correlations were observed between goal-seeking and both BDI-II and SBQ-R scores, with negative correlations between BDI-II and agreeableness and between SBQ-R and extroversion. A moderate positive correlation was noted between BDI-II and SBQ-R (ρ = 0.47, p < 0.001). Detailed findings are presented in **Figure 2**.

**Figure 3:**
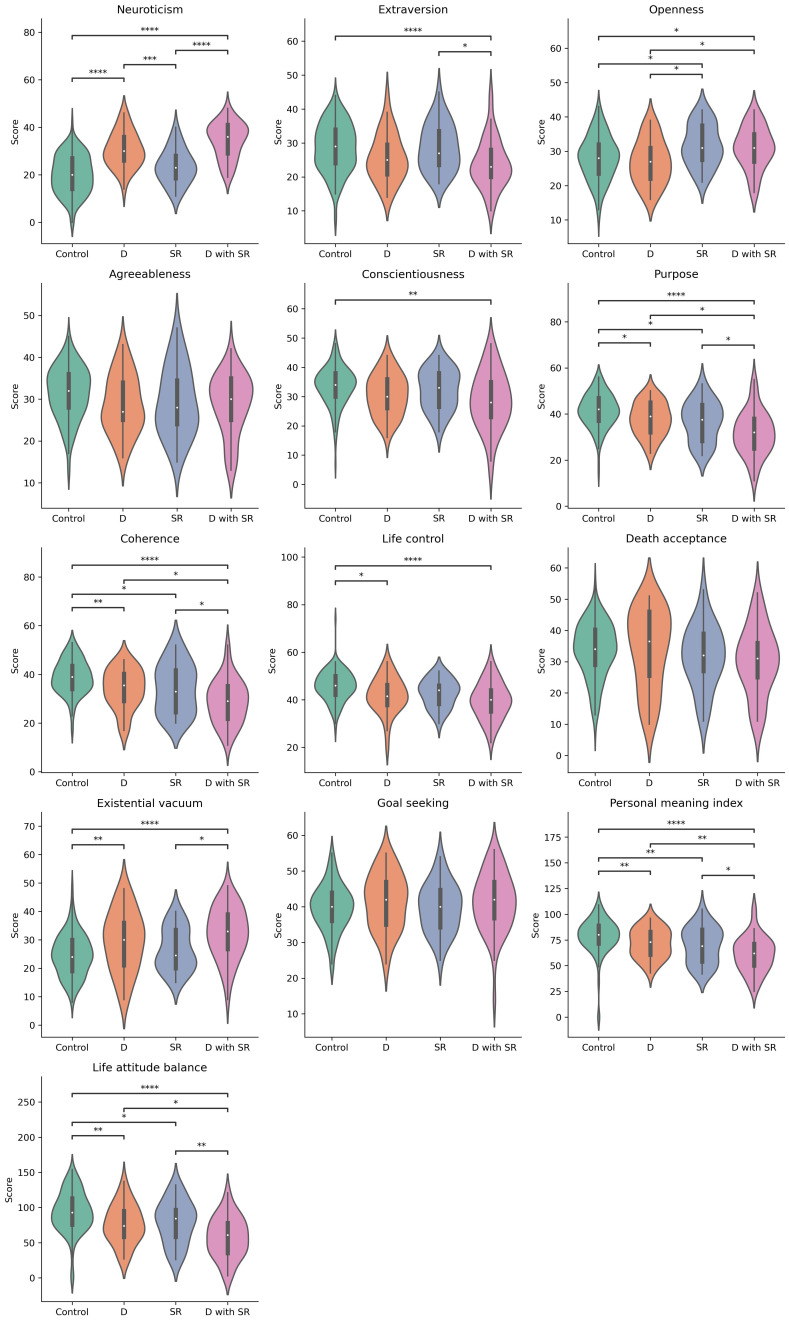
Violin plots represents result of each feature of NEO-FFI and LAP-R questionnaire in particular groups of students. The brackets indicate statistically significant differences between groups in post-hoc analysis, where * stand for padj<0,05, ** for padj<0,01, *** for padj<0,001, and **** for padj<0,0001. There was not statistically significant difference in Goal seeking in Krusal-Walis test, whereas None of group differ significantly in Agreeableness in post-hoc analysis.

Finally, we compared the features of NEO-FFI and LAP-R among the four previously distinguished groups. Students from D and D and SR groups, exhibited higher neuroticism scores compared to those with suicide risk alone (SR) and the control group. In terms of extroversion, the control and SR groups scored higher compared to the D and SR group. Participants with SR and those with D and SR had higher openness scores compared to the D and control groups. D and SR group obtained statistical lower score then control group in the terms of conscientiousness and purpose. In terms of purpose there is also a low difference between the groups: control and D, control and SR, D and D and SR, SR and D and SR. Participants in D, SR and D and SR groups has lower coherence score compared to control. Moreover students with D and SR has lower coherence score compared to those with D and SR only. In life control score, participants in D and D with Sr group has significant lower score then SR and control group. Furthermore D group has higher score in that terms then control group. The conditions: personal meaning index and life attitude balance in the control group achieved significantly higher values compared to all other groups. Similarly, the D and SR groups have higher scores in both of these indices compared to D and SR. There is no correlation between any of the groups in agreeableness, death acceptance and goal seeking. The described dependencies are presented in **Figure 3**.

## 4 Discussion

In the population studied by our team, the prevalence of depressive disorders measured by the BDI scale amounted to 32.9%; 14.5% of the patients were found to have mild depressive symptoms, 11.2% - moderate depressive symptoms, and 7.2% - severe depressive symptoms. Data available in the global literature indicate that the clinically significant severity of depressive symptoms in the group of medical students may range from approximately 10% to as much as approximately 56% (26–38). Such large differences in the obtained results are associated with the use of different psychometric tools, different versions of the BDI questionnaire, the vast majority of which are adapted to a particular population, as well as different cut-off points depending on the severity of the symptoms presented. High percentages of people with depressive symptoms were usually associated with a group with mild depressive symptoms. However, our results are consistent with the results of the meta-analysis by Puthran et al. where the prevalence of depressive symptoms was 28% (6), as well as with the results of the meta-analysis by Rotenstein et al. where the prevalence was 27.2% (7). It should also be noted that regardless of the adopted research methodology, the severity of depressive symptoms among students is much higher than among the general population. According to the representative survey of the Polish population EZOP II, conducted in 2017-2020, the prevalence of depressive disorders was 3.85%, while this percentage in the world is 3.6%, in Central Europe 3.45%, and in the European Union 4.6% (39, 40).

Our study, as well as numerous others, have demonstrated an association between Neuroticism and depressive disorders. Additionally, the subjects with depressive symptoms compared to the healthy group, had lower scores in Conscientiousness, Extroversion and Agreeableness. Traits like Extroversion and Conscientiousness often correlate with better psychological resilience (41–43).

In our study, 26.4% of participants had a significant suicide risk. Determining suicide risk in clinical practice is very difficult. Studies often take into account the presence of suicidal ideation or a history of suicide attempts which does not constitute a reliable assessment of suicide risk. Garg and co-workers in a study of 2022 reported that the prevalence of suicidal behaviors (SBQ-R > 6) among medical students was found to be 19.6% (44). A 2015 review of studies from 13 countries found the prevalence of suicidal ideation ranging from 1.8% to 53.6% (45). A 2015 systematic review reported suicidal ideation rates ranging from 4.4% to 23.1% and those of suicidal attempts ranging from 0.0% to 6.4% (46). The moderate positive correlations between Beck and SBQ-R are not surprising, and suicidal thoughts are one of the significant symptoms in the course of mood disorders (47). Although we did not observe such a significant correlation in the case of significant suicide risk as in the case of BDI and Neuroticism, at the level of intergroup comparison there are similar differences: the participants classified with significant suicide risk had a higher score in Neuroticism and lower scores in Extroversion, Conscientiousness, Agreeableness. Given that people with high Neuroticism scores tend to be less stable and more emotionally reactive, especially in response to negative events, it is natural that Neuroticism significantly increases the risk of suicide (48–50). Higher scores on the openness scale are associated with the risk of earlier depression (51). In our study, openness is associated with a higher risk of suicide. These are observations worthy of clinical attention. In students group Khosravi and Kasaeiyan showed that the occurrence of suicidal thoughts correlates with the intensity of the trait of neuroticism (52).

Unfortunately, there are few studies using the LAP-R questionnaire. We did not find any studies in which the LAP-R was compared with the BDI-II or SBQ-R in a group of students. There are studies in which the LAP-R questionnaire was validated on a group of students, but such studies do not allow us to compare the results with our research. In the study by Sipowicz et al. among people hospitalized due to SARS-CoV-2 infection, the group of people not hospitalized due to SARS-CoV-2 infection differed in the lack of a statistically significant correlation between BDI and Death Acceptance. On the other hand, in the group of people who had never been infected with the virus, no significant correlation was found between BDI and Coherence and Death Acceptance (23). However, the participants of our study classified with depressive symptoms had higher scores in Existential Vacuum; lower scores in Life Attitude Balance, Coherence, Personal Meaning Index, Purpose and Life Control. The convergence of results allows us to draw similar conclusions.

According to our widest knowledge, there are no studies using SBQ-R and LAP-R simultaneously, and therefore the presented results should be treated as pilot ones. Moderate negative correlations were observed between Coherence and Life Attitude Balance. Significantly higher scores on the Death Acceptance scale and BDI-II scores were observed in the group of people with high suicidal risk compared to healthy people. Taking into account the good correlation between the severity of depressive symptoms and the suicidal risk determined by SBQ-R, the validity of the above observations can be assumed. We found no studies that compared personality profiles between groups of patients with suicidal risk and depressive symptoms or both.

## 5 Limitation

The relatively small sample size increases the risk of selection bias and may affect the final result, therefore it may only reflect the tendencies of some medical students. Moreover, the medical students in Poland may be in a different mental state condition in comparison with students from other countries, which may not reflect the general tendency around the world. The results are also not reflective at other medical schools, as the study was carried out only at the Medical University of Łódź. The participants were students of medical sciences only, so the patterns among students of other courses remain unknown.

In our study, we used anonymous surveys that participants completed independently. This means that we did not assess the mental state of students. Based on the questionnaires we selected, we cannot make a diagnosis of a specific mental disorder; this requires a personal examination by a psychiatrist, which went beyond the objectives of the study.

During the collection of surveys and calculation of their results, surveys that were not completed in their entirety were not included. We have implemented such a strategy to limit the number of errors and obtain the most reliable results.

Assessing personality traits in the context of survey research is difficult. The results of the research are influenced by the mental condition of the person completing the survey. The fact that the patients had depressive symptoms might affect the answer to a personality long questionnaire. A certain description of personality traits requires a psychological examination and is not possible in the conditions of an anonymous survey.

Assessing suicide risk in clinical practice is difficult. Currently, there is no questionnaire that has been shown to have sensitivity and specificity based on population-based observational studies. We do not know what percentage of patients who complete a given questionnaire actually attempt suicide.

In terms of the sense of meaning in life among students from groups D, SR and D with SR, based on our study, we are unable to assess the cause-effect sequence. Determining what is the cause and what is the effect requires further research.

The study did not include details of the patients’ prior treatment.

## 6 Conclusion

People with depressive symptoms, suicide risk and both of these variables simultaneously differed in terms of personality profile and components influencing the meaning of life. People with depressive symptoms and suicide risk showed significantly greater differences in traits such as extroversion, conscientiousness and components influencing the sense of meaning in life (such as purpose, coherence, life control, existential vacuum) compared to the control group. In the case of participants from groups D and SR, these differences were not present or were less statistically significant. The fact that there are differences between people with depressive symptoms, suicide risk and both of these factors simultaneously may have a significant impact on diagnosing patients in the future. Studying the structure of personality in patients may help to estimate the risk of suicide in depressed patients. This requires further research in a clinical group, after a thorough assessment of the mental state. Considering also that personality traits are stable traits, mainly genetically determined, the occurrence of differences between specific groups may have an impact on the effectiveness of the applied treatment and further prognosis. This requires further research.

Reduced values ​​in the components of the sense of meaning in life scale in students with depressive symptoms, suicidal risk and both of these conditions at the same time may have additional clinical implications. Therapeutic interventions based on building a sense of meaning in life may bring positive results in this group of people, but this requires further research in a clinical group.

## Data Availability

The raw data supporting the conclusions of this article will be made available by the authors, without undue reservation.

## References

[1] Kötter T Pohontsch NJ Voltmer E. Stressors and starting points for health-promoting interventions in medical school from the students’ perspective: a qualitative study. Perspect Med Educ. (2015) 4:128–35. doi: 10.1007/s40037-015-0189-5 PMC4456465 26032519

[2] Messiaen M Duba A Boulangeat C Boucekine M Bourbon A Viprey M. Repeated bullying at the workplace in medical students and young doctors: the MESSIAEN national study. Eur Arch Psychiatry Clin Neurosci. (2021) 271:1123–31. doi: 10.1007/s00406-020-01144-9 32462290

[3] Dahlin M Joneborg N Runeson B. Stress and depression among medical students: a cross-sectional study. Med Educ. (2005) 39:594–604. doi: 10.1111/j.1365-2929.2005.02176.x 15910436

[4] Haldorsen H Bak NH Dissing A Petersson B. Stress and symptoms of depression among medical students at the University of Copenhagen. Scand J Public Health. (2014) 42:89–95. doi: 10.1177/1403494813503055 23999855

[5] Wallace BE Masiak J Pabis MR. Depression in medical students: reviewing its prevalence, risk factors, consequences, and management in order to provide student. Zdr Publ. (2013) 123:259–64. doi: 10.12923/j.0044-2011

[6] Puthran R Zhang MWB Tam WW Ho RC. Prevalence of depression amongst medical students: a meta-analysis. Med Educ. (2016) 50:456–68. doi: 10.1111/medu.2016.50.issue-4 26995484

[7] Rotenstein LS Ramos MA Torre M Segal JB Peluso MJ Guille C. Prevalence of depression, depressive symptoms, and suicidal ideation among medical students: A systematic review and meta-analysis. JAMA. (2016) 316:2214. doi: 10.1001/jama.2016.17324 27923088 PMC5613659

[8] Bachmann S. Epidemiology of suicide and the psychiatric perspective. Int J Environ Res Public Health. (2018) 15:1425. doi: 10.3390/ijerph15071425 29986446 PMC6068947

[9] Courtet P Nobile B Guillaume S Olié E. An urgent need for rapid anti-suicidal drugs. French J Psychiatry. (2020) 1:1–4. doi: 10.1016/j.fjpsy.2020.02.003

[10] Inskip H Harris C Barraclough B. Lifetime risk of suicide for affective disorder, alcoholism and schizophrenia. Br J Psychiatry. (1998) 172:35–7. doi: 10.1192/bjp.172.1.35 9534829

[11] Asselmann E Kunas SL Wittchen HU Martini J. Maternal personality, social support, and changes in depressive, anxiety, and stress symptoms during pregnancy and after delivery: A prospective-longitudinal study. Cimino S, editor. PloS One. (2020) 15:e0237609. doi: 10.1371/journal.pone.0237609 32833975 PMC7446870

[12] Bagby RM Joffe RT Parker JDA Kalemba V Harkness KL. Major depression and the five-factor model of personality. J Pers Disord. (1995) 9:224–34. doi: 10.1521/pedi.1995.9.3.224

[13] Michalska-Leśniewicz M Gruszczyński W. Czynniki psychologiczne w depresji. Psychiatria. (2010) 7:95–103. 20919504

[14] Carstens JA Spangenberg JJ. Major depression: A breakdown in sense of coherence? Psychol Rep. (1997) 80:1211–20. doi: 10.2466/pr0.1997.80.3c.1211 9246887

[15] Choenarom C Williams RA Hagerty BM. The role of sense of belonging and social support on stress and depression in individuals with depression. Arch Psychiatr Nursing. (2005) 19:18–29. doi: 10.1016/j.apnu.2004.11.003 15765368

[16] Maryam Hedayati MA Mahmoud Khazaei MA. An investigation of the relationship between depression, meaning in life and adult hope. Proc - Soc Behav Sci. (2014) 114:598–601. doi: 10.1016/j.sbspro.2013.12.753

[17] Beck AT Steer RA Brown G. Beck depression inventory–II. psychol Assess. (1996). doi: 10.1037/t00742-000

[18] Segal DL Coolidge FL Cahill BS O’Riley AA. Psychometric properties of the beck depression inventory—II (BDI-II) among community-dwelling older adults. Behav Modif. (2008) 32:3–20. doi: 10.1177/0145445507303833 18096969

[19] Wang YP Gorenstein C. Psychometric properties of the Beck Depression Inventory-II: a comprehensive review. Braz J Psychiatry. (2013) 35:416–31. doi: 10.1590/1516-4446-2012-1048 24402217

[20] McCrae RR Costa PT Jr. A contemplated revision of the NEO Five-Factor Inventory. Pers Individ Differ. (2004) 36:587–96. doi: 10.1016/S0191-8869(03)00118-1

[21] Zawadzki B Strelau J Szczepaniak P Śliwińska M. Inwentarz osobowości NEO-FFI Costy i McCrae. Adaptacja polska Podręcznik, Warszawa: Pracownia Testów Psychologicznych PTP (1998).

[22] Reker GT. The Life Attitude Profile-Revised:(LAP-R). Ontario, Canada: Student Psychologists Press Peterborough (2001).

[23] Sipowicz K Pietras T Mosiołek A Sobstyl M Ring M Kamecki K. The sense of loneliness and meaning in life in post-COVID convalescents—a preliminary study. Front Psychiatry. (2023) 14:1296385. doi: 10.3389/fpsyt.2023.1296385 38188044 PMC10768000

[24] Chodkiewicz J Gruszczyńska E. The Polish adaptation of the Suicide Behaviors Questionnaire-Revised by A. Osman et al. Psychiatria Polska. (2020) 54:101–11. doi: 10.12740/PP/OnlineFirst/93492 32447359

[25] Osman A Bagge CL Gutierrez PM Konick LC Kopper BA Barrios FX. The Suicidal Behaviors Questionnaire-Revised (SBQ-R): validation with clinical and nonclinical samples. Assessment. (2001) 8:443–54. doi: 10.1177/107319110100800409 11785588

[26] Bartosik NK Frankowski R Kobierecki M Deska K Twarowski A Bąk B. The association between affective temperaments and depressive symptoms in a population of medical university students, Poland. Front Psychiatry. (2023) 14:1077940. doi: 10.3389/fpsyt.2023.1077940 37065892 PMC10098149

[27] Burger PHM Scholz M. Gender as an underestimated factor in mental health of medical students. Ann Anat - Anatomischer Anzeiger. (2018) 218:1–6. doi: 10.1016/j.aanat.2018.02.005 29551695

[28] Džubur A Abdulahović D Kurspahić-Mujčić A Džubur A Loga-Zec S Škrijelj V. Depressive symptoms among sarajevo university students: prevalence and socio-demographic correlations. ama. (2018) 47:155. doi: 10.5644/ama2006-124.227 30585067

[29] Fitzpatrick O Biesma R Conroy RM McGarvey A. Prevalence and relationship between burnout and depression in our future doctors: a cross-sectional study in a cohort of preclinical and clinical medical students in Ireland. BMJ Open. (2019) 9:e023297. doi: 10.1136/bmjopen-2018-023297 PMC6502268 31048421

[30] Jaroszewska AA Tyras S Dziewit M Jaroszewska J Podhorodecka K. Prevalence of anxiety and depression among domestic and foreign medical students in Poland. Pol Ann Med. (2020) 27(2):135–7. doi: 10.29089/2020.20.00125

[31] Kożybska M Kurpisz J Radlińska I Skwirczyńska E Serwin N Zabielska P. Problematic Internet Use, health behaviors, depression and eating disorders: a cross-sectional study among Polish medical school students. Ann Gen Psychiatry. (2022) 21:5. doi: 10.1186/s12991-022-00384-4 35148793 PMC8832421

[32] Mancevska S Bozinovska L Tecce J Pluncevik-Gligoroska J Sivevska-Smilevska E. Depression, anxiety and substance use in medical students in the Republic of Macedonia. Bratisl Lek Listy. (2008) 109:568–72. 19348380

[33] Mokros Ł Witusik A Michalska J Panek M Nowakowska-Domagała K. Sleep quality, chronotype, temperament and bipolar features as predictors of depressive symptoms among medical students. Chronobiol Int. (2017) 34:708–20. doi: 10.1080/07420528.2017.1316730 28488895

[34] Oleszko A Szczepańska E Janion K Jośko-Ochojska J. Nutrition behaviours and the occurrence of depressive symptoms among the students in the institutions of higher education in Silesia (Poland). Rocz Panstw Zakl Hig. (2019) 70(1):69–77. doi: 10.32394/rpzh 30837748

[35] Seweryn M Tyrała K Kolarczyk-Haczyk A Bonk M Bulska W Krysta K. Evaluation of the level of depression among medical students from Poland, Portugal and Germany. Psychiatr Danub. (2015) 27 Suppl 1:S216–222. 26417766

[36] Silva V Costa P Pereira I Faria R Salgueira AP Costa MJ. Depression in medical students: insights from a longitudinal study. BMC Med Educ. (2017) 17:184. doi: 10.1186/s12909-017-1006-0 29017594 PMC5633876

[37] Zalewska A Gałczyk M Sobolewski M Fernandes H. A pilot cross-sectional study on the level of depression and physical activity among students in Poland and Portugal in the second year of the COVID-19 pandemic. JCM. (2023) 12:2541. doi: 10.3390/jcm12072541 37048625 PMC10095387

[38] Žaludek A Fialová A Pokorná K Hudáč P David J Marx D. Comparison of prevalence of depression symptoms and history of suicidality in students of medical schools and other study programmes of Charles University. Cent Eur J Public Health. (2023) 31:217–22. doi: 10.21101/cejph.a7680 37934486

[39] Institute for Health Metrics and Evaluation. IHME (2021). Available online at: http://www.healthdata.org (accessed November 12, 2021).

[40] Institute of Psychiatry and Neurology. Comprehensive Study of the State of Mental Health of the Society and Its Conditions (EZOP II) (2021). Available online at: https://ezop.edu.pl/wyniki-badania/ (accessed January 29, 2022).

[41] Lica MM Papai A Salcudean A Crainic M Covaciu CG Mihai A. Assessment of psychopathology in adolescents with insulin-dependent diabetes (IDD) and the impact on treatment management. Children. (2021) 8:414. doi: 10.3390/children8050414 34069480 PMC8159087

[42] Nieto M Visier ME Silvestre IN Navarro B Serrano JP Martínez-Vizcaíno V. Relation between resilience and personality traits: The role of hopelessness and age. Scandinavian J Psychol. (2023) 64:53–9. doi: 10.1111/sjop.12866 PMC10087311 36057793

[43] Rosellini AJ Brown TA. The NEO five-factor inventory: latent structure and relationships with dimensions of anxiety and depressive disorders in a large clinical sample. Assessment. (2011) 18:27–38. doi: 10.1177/1073191110382848 20881102 PMC5639474

[44] Garg S Chauhan A Singh S Bansal K. Epidemiological risk factors of suicidal behavior and effects of the components of coping strategies on suicidal behavior in medical students: A North-Indian institution-based cross-sectional study. J Neurosci Rural Pract. (2022) 13:382–92. doi: 10.1055/s-0042-1744225 PMC9357490 35946011

[45] Coentre R Góis C. Suicidal ideation in medical students: recent insights. AMEP. (2018) 9:873–80. doi: 10.2147/AMEP.S162626 PMC6276609 30568525

[46] Moutinho Coentre R Luisa Figueira M. Depression and suicidal behavior in medical students: a systematic review. Curr Psychiatry Rev. (2015) 11:86–101. doi: 10.2174/1573400510666140807005141

[47] Harmer B Lee S Rizvi A Saadabadi A. Suicidal ideation. In: StatPearls. StatPearls Publishing, Treasure Island (FL (2024). Available at: http://www.ncbi.nlm.nih.gov/books/NBK565877/. 33351435

[48] Chan SWY Goodwin GM Harmer CJ. Highly neurotic never-depressed students have negative biases in information processing. Psychol Med. (2007) 37:1281–91. doi: 10.1017/S0033291707000669 17493298

[49] Paulus DJ Vanwoerden S Norton PJ Sharp C. Emotion dysregulation, psychological inflexibility, and shame as explanatory factors between neuroticism and depression. J Affect Disord. (2016) 190:376–85. doi: 10.1016/j.jad.2015.10.014 26546773

[50] Servaas MN van der Velde J Costafreda SG Horton P Ormel J Riese H. Neuroticism and the brain: A quantitative meta-analysis of neuroimaging studies investigating emotion processing. Neurosci Biobehav Rev. (2013) 37:1518–29. doi: 10.1016/j.neubiorev.2013.05.005 23685122

[51] Koorevaar AML Comijs HC Dhondt ADF van Marwijk HWJ van der Mast RC Naarding P. Big Five personality and depression diagnosis, severity and age of onset in older adults. J Affect Disord. (2013) 151:178–85. doi: 10.1016/j.jad.2013.05.075 23820093

[52] Khosravi M Kasaeiyan R. The relationship between neuroticism and suicidal thoughts among medical students: Moderating role of attachment styles. J Family Med Prim Care. (2020) 9:2680–7. doi: 10.4103/jfmpc.jfmpc_1200_19 PMC7491799 32984107

